# Supplementary material for: Fat Loss in Patients with Metastatic Clear Cell Renal Cell Carcinoma Treated with Immune Checkpoint Inhibitors
Source: Int J Mol Sci. 2023 Feb 16;24(4):3994. doi: 10.3390/ijms24043994 (PMC9967473; doi:10.3390/ijms24043994)
Supplement: Supplementary file 1 [file ijms-24-03994-s001.zip › ijms-2199151-supplementary.pdf]

Fat loss in Patients with Metastatic Clear Cell Renal Cell Carcinoma Treated with Immune Checkpoint Inhibitors

Supplemental Material

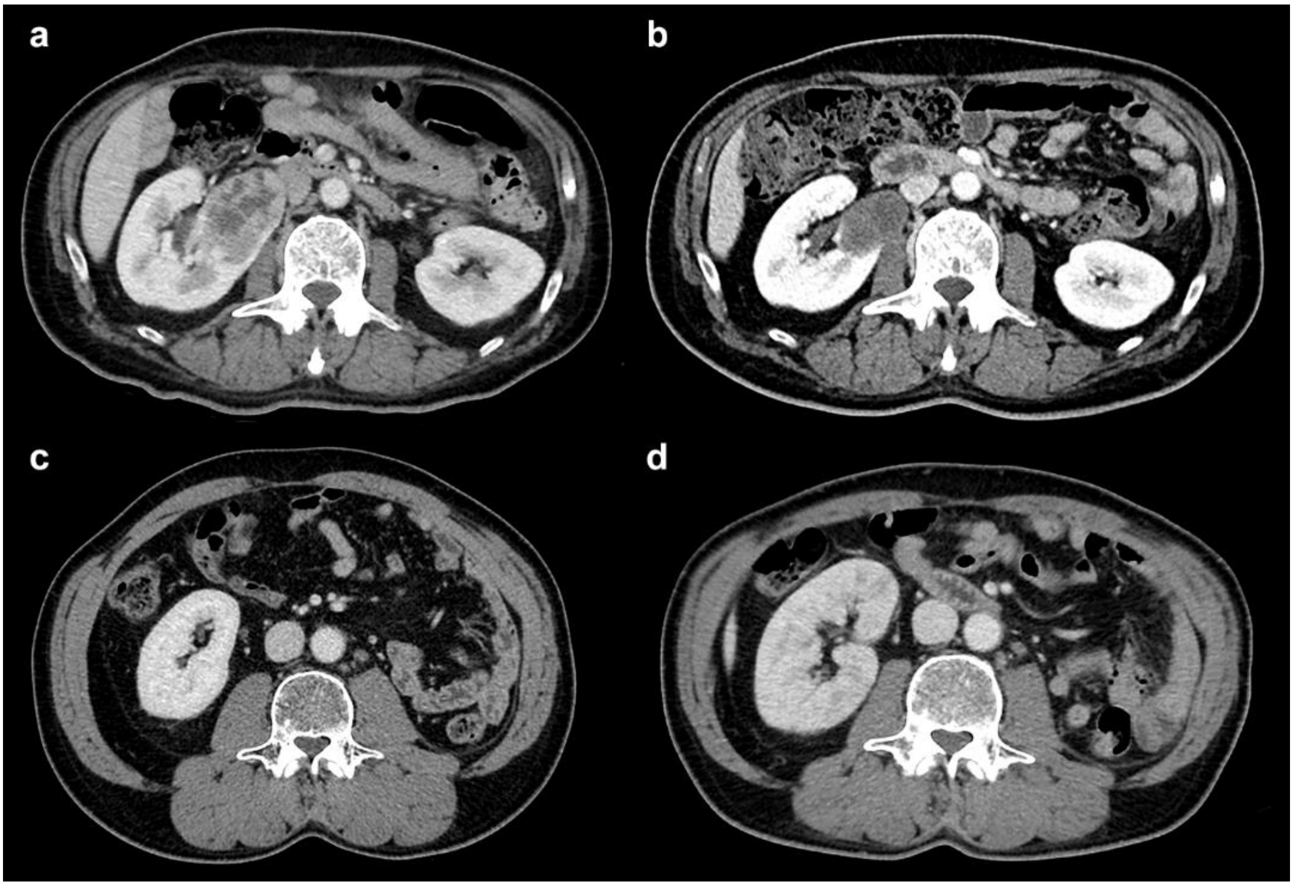

Supplemental Figure S1. Representative CT images showing body composition changes in two patients with metastatic clear cell renal cell carcinoma

Pretreatment (a) and posttreatment CT images after 75 days (b) of a 65-year-old man who achieved a partial response. The cross-sectional areas of subcutaneous fat, visceral fat, and total fat changed from 99.9 cm^2, 77.5 cm^2, and 177.4 cm^2 to 105.7 cm^2, 85.2 cm^2, and 190.9 cm^2, respectively: these changes were not significant. Pre-treatment (c) and post-treatment CT images after 70 days (d) of a 52-year-old man who exhibited progressive disease after four months. The cross-sectional areas of subcutaneous fat, visceral fat, and total fat substantially decreased from 110.6 cm^2, 150.5 cm^2, and 261.0 cm^2 to 80.7 cm^2, 89.9 cm^2, and 170.6 cm^2, respectively. CT, computed tomography.

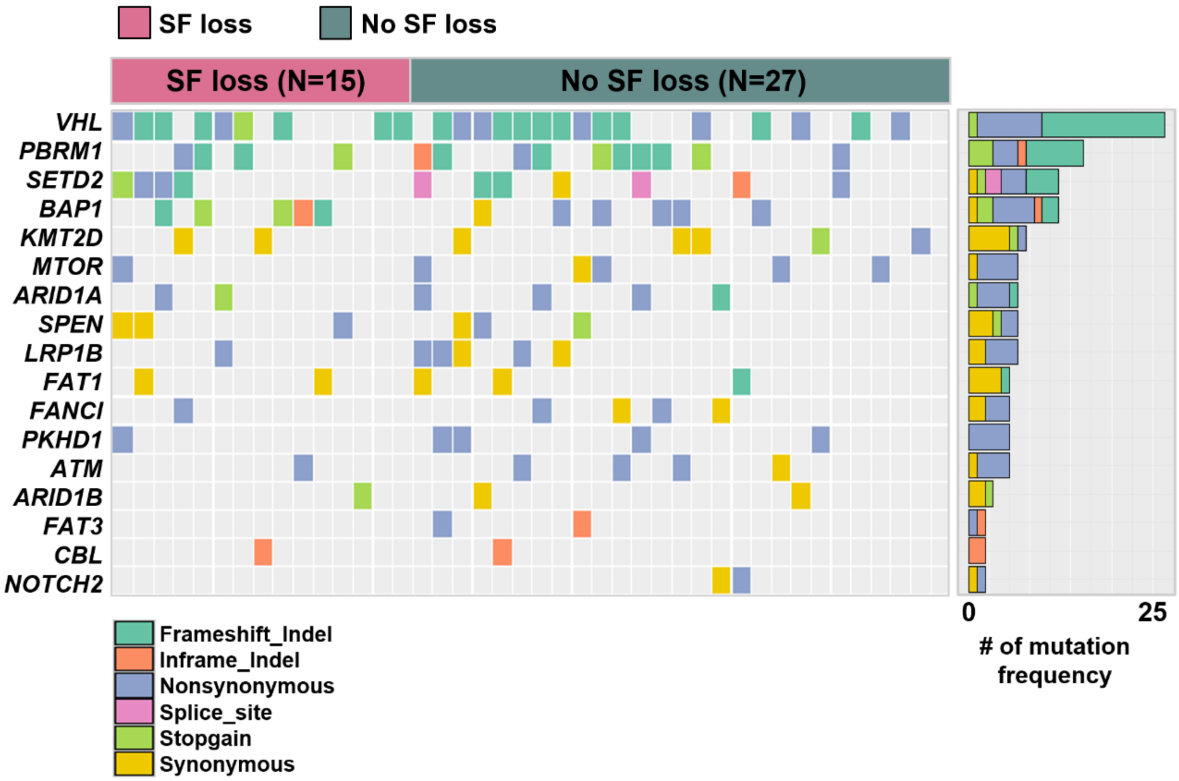

Supplemental Figure S2. Overall mutational landscape in patients with clear cell renal cell carcinoma with and without SF loss

A heatmap showing 17 recurrently mutated genes ordered by mutation frequency. SF, subcutaneous fat.

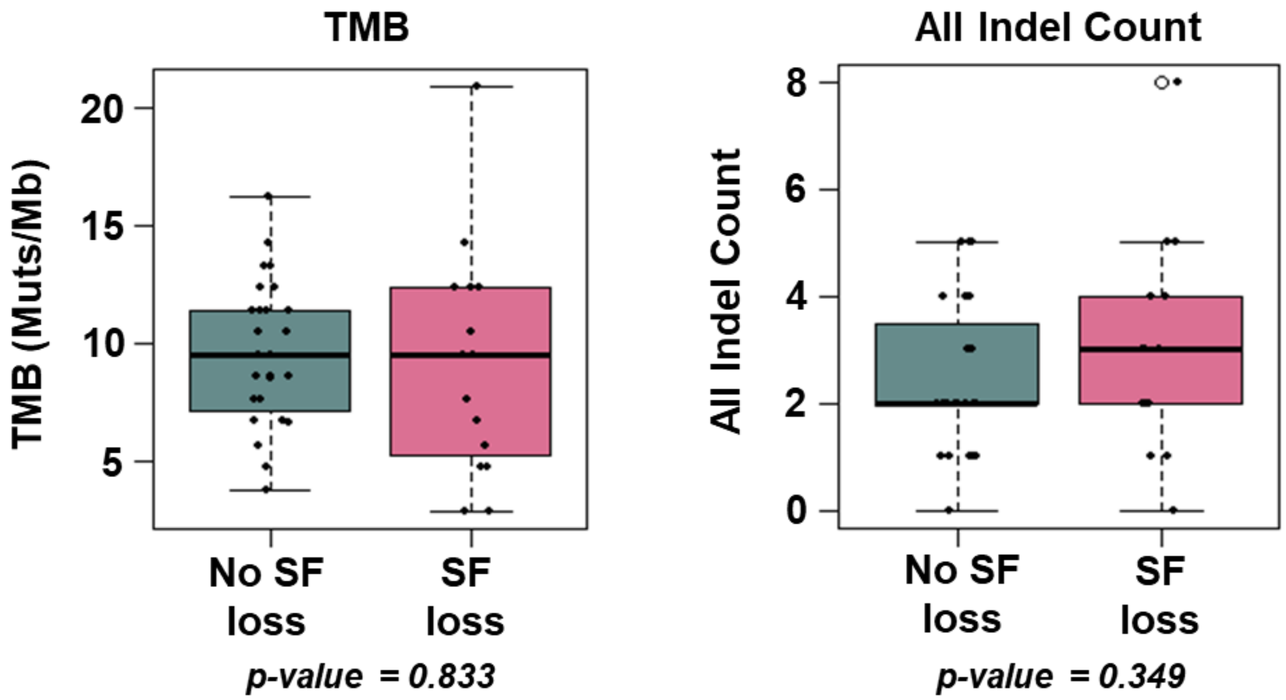

Supplemental Figure S3. Genomic alterations according to SF loss

Tumor mutational burden and total indel count between samples without and with SF loss. T-test p-value shown. Midlines and bars indicate the medians and the 5–95th percentiles, respectively. Muts, mutations; Mb, megabase; TMB, tumor mutational burden; SF, subcutaneous fat.

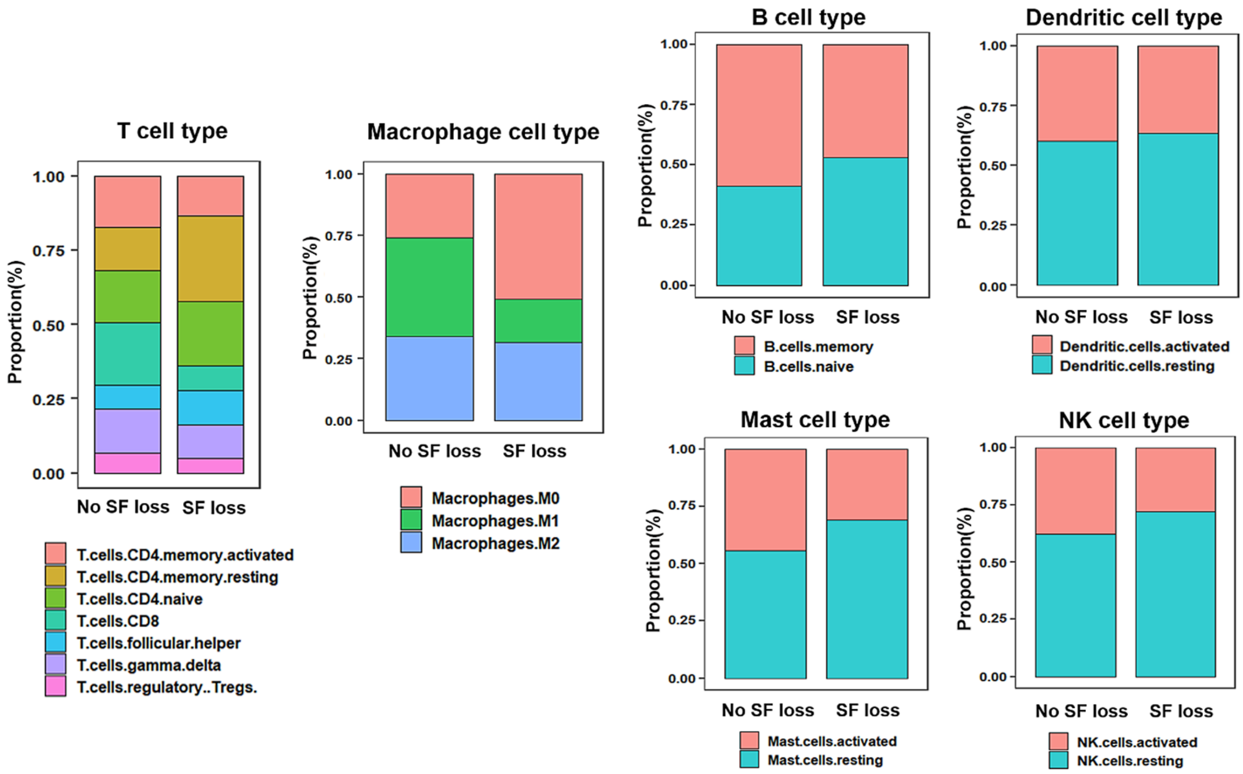

Supplemental Figure S4. Proportion of diverse immune cell subpopulations according to SF loss

CIBERSORTx findings showing a comparison of the percentage of distinct immune cell subpopulations (T cells, macrophages, B cells, dendritic cells, mast cells, and NK cells) according to SF loss. SF, subcutaneous fat.

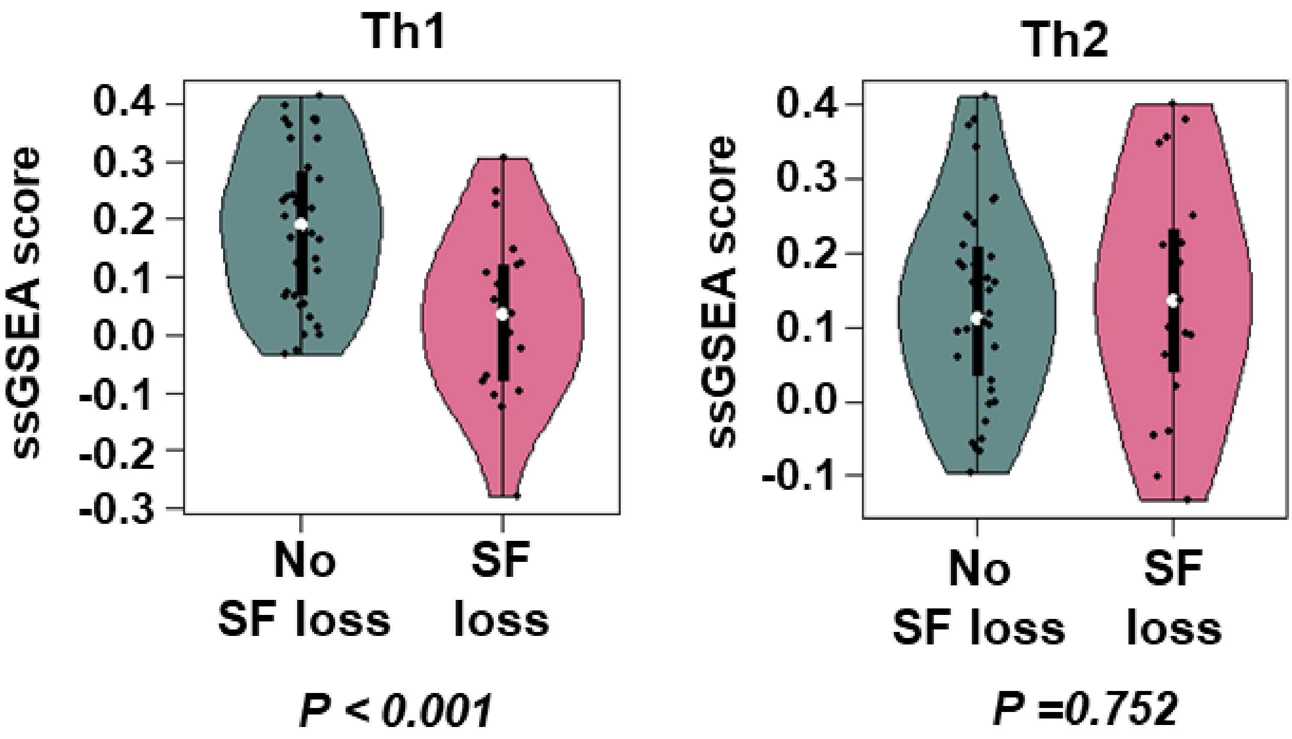

Supplemental Figure S5. ssGSEA analysis using the gene signatures of Th1 and Th2

Violin plots showing ssGSEA scores using the gene signatures of Th1 and Th2 from Bindea *et al.* in groups with and without SF loss. SF, subcutaneous fat; ssGSEA, single-sample gene set enrichment test.

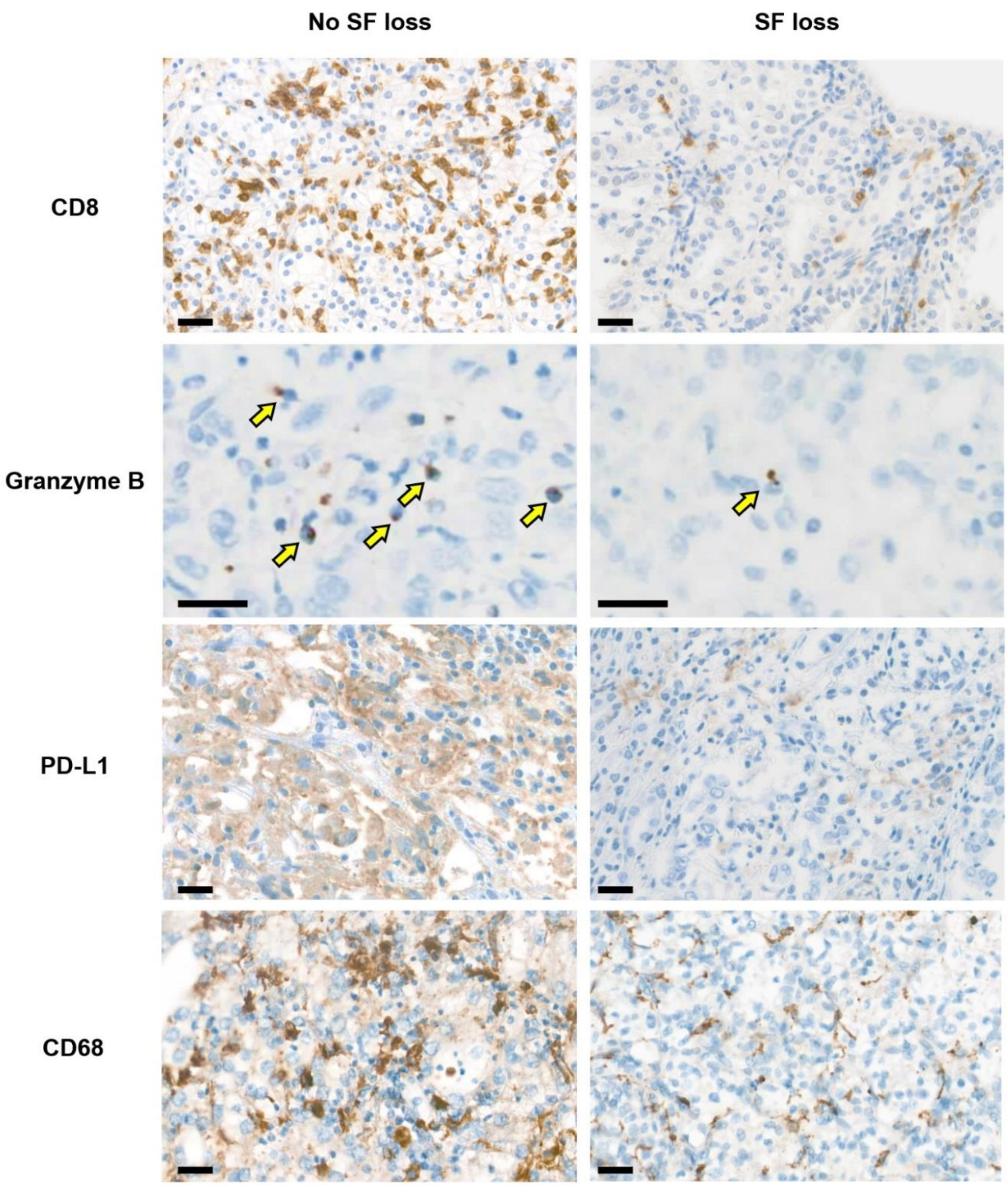

Supplemental Figure S6. Immune cell population characterized by IHC in patients without SF loss and patients with SF loss

Representative IHC images of CD8, granzyme B, PD-L1, and CD68 staining in patients without SF loss (*left*) and patients with SF loss (*right*). Granzyme B+ cells are indicated by yellow arrows. Scale bars in the micrographs indicate 25 μm. IHC, immunohistochemistry; SF, SF loss.

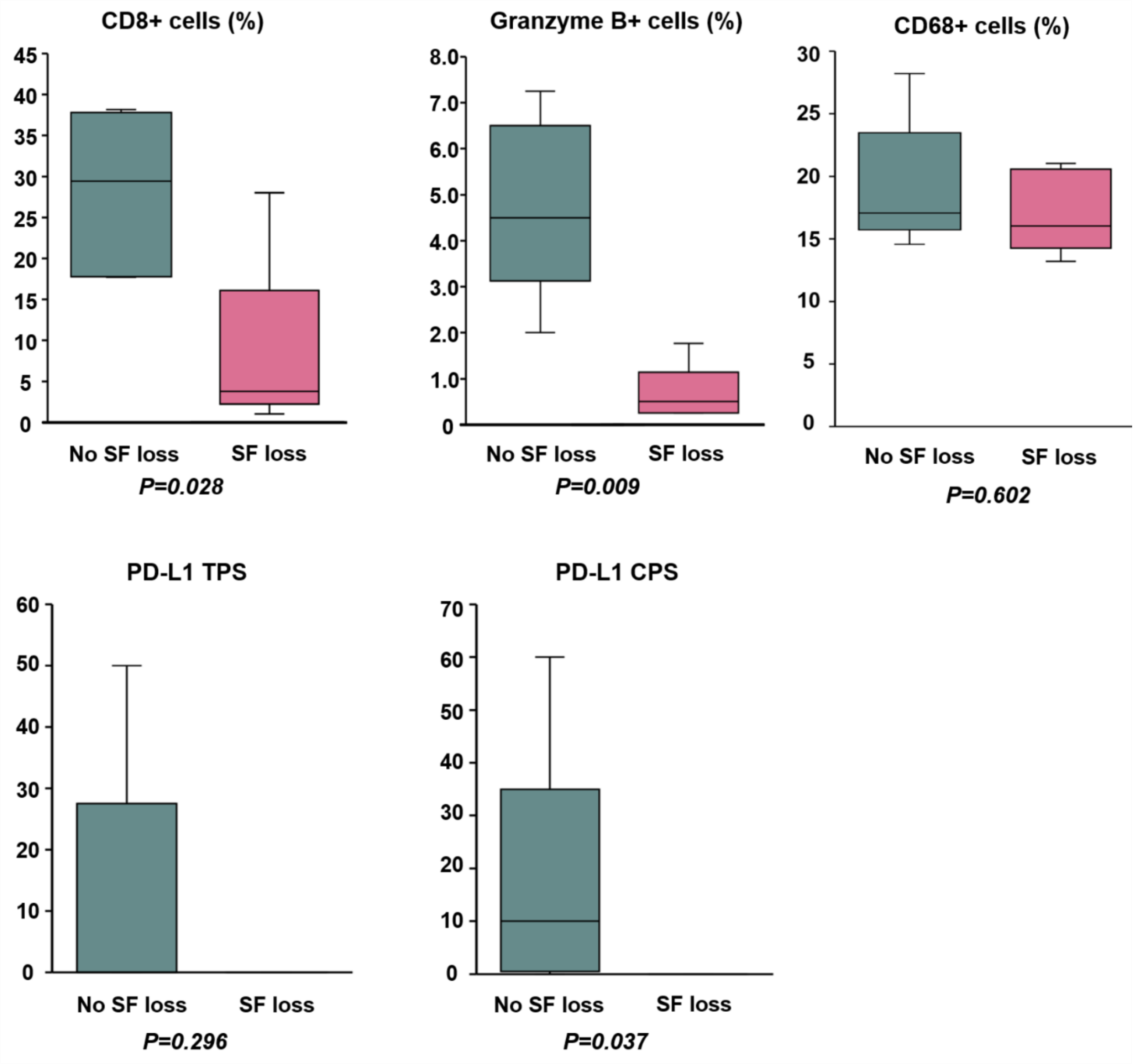

Supplemental Figure S7. Quantification of CD8, Granzyme B, PD-L1, and CD68 expressing cells in representative samples of patients without SF loss (*n* = 5) and patients with SF loss (*n* = 5)

Midlines and bars indicate the medians and the 5–95th percentiles, respectively. SF, subcutaneous fat loss; TPS, tumor proportional score; CPS, combined positive score.

Supplemental Table S1. Objective response rate, best overall response, and response group stratified by subcutaneous fat loss.

**Variables**	**SF loss † (*n* = 20)**	**No SF loss (*n* = 40)**	***p***
Objective response rate, % (95% CI) *	10.0% (1.2–31.7%)	62.5% (45.8–77.3%)	<0.001
Best overall response *			<0.001
Complete response, *n* (%) *	0 (0.0%)	6 (15.0%)	0.165
Partial response, *n* (%) *	2 (10.0%)	19 (47.5%)	0.004
Stable disease, *n* (%) *	2 (10.5%)	7 (17.5%)	0.704
Progressive disease, *n* (%) *	15 (75.0%)	8 (20.0%)	<0.001
Unable to determine, *n* (%)	1 (0.5%)	0 (0.0%)	–
Response group *			<0.001
Clinical benefit, *n* (%) *	2 (10.0%)	25 (62.5%)	<0.001
Intermediate benefit, *n* (%) *	2 (10.0%)	5 (12.5%)	1.000
No clinical benefit, *n* (%) *	15 (75.0%)	10 (25.0%)	<0.001
Unable to determine, *n* (%)	1 (0.5%)	0 (0.0%)	–

CI: confidence interval; SF: subcutaneous fat.

* Fisher's exact test.

† Defined as ΔSF < -5 %/month.

Supplemental Table S2. Multivariable models for interaction of fat loss with line of treatment and IMDC risk criteria.

Characteristic	Line of treatment		*p* for interaction	IMDC risk criteria		*p* for interaction
	First line	Non-first line		Favorable / intermediate	Poor	
	Adjusted HR † (95% CI)	Adjusted HR † (95% CI)		Adjusted HR ‡ (95% CI)	Adjusted HR ‡ (95% CI)	
**OS**						
ΔSF *	2.32 (0.61–8.81)	1.42 (0.98–2.04)	0.926	1.41 (0.89–2.25)	2.11 (0.82–5.40)	0.968
ΔVF *	1.12 (0.85–1.47)	1.17 (0.96–1.43)	0.631	1.11 (0.84–1.46)	1.17 (0.93–1.47)	0.894
ΔTF *	1.41 (0.72–2.75)	1.38 (0.96–1.99)	0.710	1.33 (0.83–2.14)	1.42 (0.88–2.28)	0.490
**PFS**						
ΔSF *	2.84 (1.30–6.24)	1.52 (1.09–2.10)	0.902	1.85 (1.20–2.86)	2.67 (0.95–7.50)	0.581
ΔVF *	1.12 (0.90–1.38)	1.11 (0.99–1.25)	0.570	1.15 (0.96–1.38)	1.13 (0.94–1.35)	0.962
ΔTF *	1.57 (0.96–2.57)	1.29 (1.03–1.61)	0.983	1.54 (1.08–2.19)	1.43 (0.95–2.16)	0.540

IMDC: International Metastatic Renal Cell Carcinoma Database Consortium; SF: subcutaneous fat; VF: visceral fat; TF: total fat; HR: hazard ratio; CI: confidence interval; OS: overall survival; PFS: progression-free survival.

* Continuously, per -5 %/month.

† Adjusted for age (≥ 65 years/<65 years), sex (male/female), ΔBW (continuously, per 1 %/month), prior nephrectomy (yes/no), ECOG performance status (≥1/0), IMCD risk criteria (favorable/intermediate/poor), and number of metastases (≥2/1).

‡ Adjusted for age (≥ 65 years/<65 years), sex (male/female), ΔBW (continuously, per 1 %/month), line of treatment (first line/non-first line), prior nephrectomy (yes/no), ECOG performance status (≥1/0), and number of metastases (≥2/1).
